# Parenting approaches and digital technology use of preschool age children in a Chinese community

**DOI:** 10.1186/1824-7288-40-44

**Published:** 2014-05-07

**Authors:** Cynthia Sau Ting Wu, Cathrine Fowler, Winsome Yuk Yin Lam, Ho Ting Wong, Charmaine Hei Man Wong, Alice Yuen Loke

**Affiliations:** 1 GH506, School of Nursing, The Hong Kong Polytechnic University, Hung Hom, Kowloon, Hong Kong, China; 2 Faculty of Health, University of Technology Sydney, Sydney, Australia; 3 Princess Margaret Hospital, Hong Kong, China

**Keywords:** Child health, Digital technologies, Internet, Parenting, Preschooler, Screen time

## Abstract

**Background:**

Young children are using digital technology (DT) devices anytime and anywhere, especially with the invention of smart phones and the replacement of desktop computers with digital tablets. Although research has shown that parents play an important role in fostering and supporting preschoolers’ developing maturity and decisions about DT use, and in protecting them from potential risk due to excessive DT exposure, there have been limited studies conducted in Hong Kong focusing on parent-child DT use. This study had three objectives: 1) to explore parental use of DTs with their preschool children; 2) to identify the DT content that associated with child behavioral problems; and 3) to investigate the relationships between approaches adopted by parents to control children’s DT use and related preschooler behavioral problems.

**Methods:**

This exploratory quantitative study was conducted in Hong Kong with 202 parents or guardians of preschool children between the ages of 3 and 6 attending kindergarten. The questionnaire was focused on four aspects, including 1) participants’ demographics; 2) pattern of DT use; 3) parenting approach to manage the child’s DT use; and 4) child behavioral and health problems related to DT use. Multiple regression analysis was adopted as the main data analysis method for identifying the DT or parental approach-related predictors of the preschooler behavioral problems.

**Results:**

In the multiple linear regression model, the ‘restrictive approach score’ was the only predictor among the three parental approaches (B:1.66, 95% CI: [0.21, 3.11], p < 0.05). Moreover, the viewing of antisocial behavior cartoons by children also significantly increased the tendency of children to have behavioral problem (B:3.84, 95% CI: [1.66, 6.02], p < 0.01).

**Conclusions:**

Since preschool children’s cognitive and functional abilities are still in the developmental stage, parents play a crucial role in fostering appropriate and safe DT use. It is suggested that parents practice a combination of restrictive, instructive and co-using approaches, rather than a predominately restrictive approach, to facilitate their child’s growth and development. Further studies are needed to explore the parent-child relationship and parents’ self-efficacy when managing the parent-child DT use, to develop strategies to guide children in healthy DT use.

## Background

Children are now born into a cyber world that will result in different experiences and opportunities. Young children are using digital technology (DT) devices anytime and anywhere, especially with the invention of smart phones and the replacement of desktop computers with digital tablets [1]. With the rapid advancements in DTs, there is an explosion of electronic media games/learning packages directed at preschool (3 to 6 years) children in many societies [2-4]. In this early part of the 21^st^ century it is anticipated that there will continue to be many new technologies developed which will impact our daily lives [1].

DT use has both positive and negative impacts on preschool children’s development in five domains. *Academically*, a positive attitude toward learning, increased alphabet recognition, improved reading skills, and enhanced early language and mathematical knowledge development attributed to DT use [5]. *Cognitively*, it is beneficial to use DT to improve visual intelligence skills and develop psychomotor skills [6]. On the other hand, DT use can negatively affect preschool age children in physical, psychological, and social aspects of development. *Physically*, preschool children with increasing DT use tend to be less physically active, increasing the risks of obesity and musculoskeletal problems [7]. *Psychologically*, spending too much time using DTs increases preschool children’s risk of developing addictive disorders, depression, and aggressive and violent behaviour, and leads them to experience difficulty in distinguishing fantasy from reality [8]. *Socially*, frequent DT use is associated with decreased family time and communication, increased social isolation, and hindered development of preschoolers’ interpersonal skills [5]. In summary, the positive impacts of DT use are found mostly in the academic and cognitive domains, while negative impacts are found mostly in the physical, psychological and social domains.

Parental modeling was defined as ‘a process of observational learning in which the parent’s behavior acts as a stimulus for similar behavior in his or her child’ [9,10]. As young children learn new skills by mimicking their parents’ use of computers and other DTs, parental education and modeling appropriate behaviors becomes a critical factor for regulating preschoolers’ safe use of DTs [11-13].

Three main parental behavior approaches (i.e. restrictive, instructive, and co-using approaches) have been identified in related literature. The ‘restrictive approach’ refers to rules being set to control children’s DT use. Parents usually put time limits on their child’s DT use and decide on program content [11,12]. The ‘instructive approach’ refers to parents providing recommendations and suggestions to enhance their child’s appropriate use of DTs [12]. Parents will provide education to their children about what programs are appropriate and explain the program’s explicit and implicit meaning [14]. The ‘co-using approach’ is also known as co-playing or parental modeling. It means parents are simply present while the child is using the medium. No purposeful discussion on the meaning or effects of content is involved but just sharing the experience from parents [11,15]. It is acknowledged as enhancing the parent-child relationship, and facilitating development of cognitive, motor and other developmental skills [12,16]. All three parental approaches are associated with the reduction in excessive DT exposure; therefore, active parental involvement could lead children to utilize DTs in beneficial ways [11,12].

According to the Hong Kong Census and Statistics Department in 2012, 80.3% of all households had personal computers at home, with 78.6% connected to the Internet. Nearly all (99.2%) children ages 10 to 14 used a computer during the 12 months before enumeration [17]. These Hong Kong statistics are consistent with estimates from other developed countries that between 70% and 90% of preschoolers use a computer at home or school [18]. According to a 2005–2006 survey conducted by the Hong Kong Department of Health, 70.3% of children ages 4 to 14 played on the computer or used video games (including accessing the Internet), with a median use of 5 days per week; 57.8% spent 2 or more hours per day watching a screen, including the time spent on watching television or videos, and using a computer or playing computer games [19]. More than 50% of children in Hong Kong were exposed to excessive DT use, as defined by the recommendation of the American Academy of Pediatrics that use be restricted to less than 2 hours per day [20].

Research on the risk of DT use, especially on children younger than 6 years of age, is needed [14,21]. There has been limited parent-child DT use studies conducted in Hong Kong. On the other hand, DTs signify unique peculiarities in different geographical locations and social groups, and cultural differences may affect parenting approaches [22,23]. It is likely in other countries that these cultural and other factors may affect parent-child DT use in different ways and existing knowledge may not always be applicable in Hong Kong [24,25].

This paper reports on the DT use patterns and behaviors of Hong Kong preschool children and their parents, and the relationship to three parenting approaches – restrictive, instructive and co-using. Several issues were identified within this study including: the time the parents spent using DTs per day with their child, and a link between the type of parenting approach and behavioral problems. In this study, the term DT refers to all kinds of digital product (e.g. television, digital tablet, smart phone, etc.), and Internet related behavioral problems are defined as personal, family, and academic problems.

## Methods

This exploratory quantitative study was conducted in the three regions of Hong Kong (Hong Kong Island, Kowloon and the New Territories). There were 3 main study objectives:

1. To explore parent’s use of DTs with their preschool children,

2. To identify the DT content that associated with child behavioral problems

3. To investigate the relationship between the approaches adopted by parents to control children’s DT use and related preschooler behavioral problems.

A questionnaire in Chinese that took approximately 15 to 20 minutes to complete was used to collect descriptive data. An information sheet and a questionnaire were distributed to every parent who met the selection criteria via the kindergarten teachers. Parents or guardians of preschool children ages 3 to 6 were asked to complete the questionnaire. Inclusion criteria were: 1) parent or guardian of a preschool child; 2) living with and caring for the preschool child; and 3) able to read Chinese. If the parent had more than one child in the age group, the parent was instructed to answer the question referring to the child that was attending kindergarten.

Parents were requested to seal the completed questionnaire in a pre-addressed envelope and place it in a sealed project box. A member of the research team collected the questionnaires from the kindergarten after a two-week period.

Implied consent was obtained if parents returned the completed questionnaire. A total of 385 questionnaires were distributed to the parents or guardians of preschool children, and 202 questionnaires were collected from the three kindergartens, with a response rate of 52.5%.

### Questionnaire

The four-part questionnaire was developed based on related literature. Parts one and two focused on a) demographics of the children, parents, and home environment; and b) pattern of parent-child and child’s DT use (including type, availability, accessibility, content, purpose and frequency/duration of parent-child and child’s DT use in the last 3 months). Part three adopted a modified version of “Parental Approach Patterns on Children’s Media Use,” which focused on the type of parenting approach used to manage a child’s DT use [11]. Questions represent the three types of parental approaches (i.e. instructive, co-using, and restrictive approaches) included: a) Instructive approach: e.g. ‘Tell my child when somebody in a games/show does something that is bad’; b) Co-using approach: e.g. ‘Having online and digital activities together with my child because it is important to her/him to do this activity together with me’; and c) Restrictive approach: e.g. ‘Not allow my child to watch/play a certain program/game’. Part four was modified from the original version of the “Parent-Child Internet Addiction Test” which focused on child behavioral and health problems related to DT use [26]. Five original questions were removed, as the behaviors (e.g. neglecting household chores, receiving strange phone calls from new on-line friends) were not relevant to preschool children [27,28]. Approval from the original authors was received for the use of the “Parental Approach Patterns on Children’s Media Use” and “Parent-Child Internet Addiction Test.”

### Validity and reliability

A professional translator first translated the instrument from English to Chinese; then another translator completed the translation back to English. Neither was familiar with the original questionnaire text. The research team and a community nurse researcher verified the consistency of the English original instrument and the translated version.

A panel of 3 experts affirmed the content validity of the instrument and a test-retest reliability assessment was conducted by inviting 12 parents who were not involved in the study to complete the questionnaires. The experts included a professor who was involved in the development of the original “Parent-Child Internet Addiction Test,” a professor who was involved in the development of the original “Parental Approach Patterns on Children’s Media Use” questionnaire, and a professor with nursing education expertise. The scale level content validity index (CVI) was calculated to be 0.989. The value was calculated by averaging the item level CVI for all questions in the scale. Amendments were completed to address the experts’ comments. The overall reliability was satisfactory, with a Pearson correlation coefficient of 0.75; the intra-correlation coefficients of the modified scale for parental approach, and for child behavioral problems, were 0.939 and 0.919 respectively.

### Ethics consideration

Ethical approval was obtained from the Human Subjects Ethics Sub-Committee. Respondents participated in the study on a voluntary basis. Confidentiality and anonymity of the subject were maintained throughout the study. All data were amalgamated to further reduce the potential for identification of parents.

### Data analysis

The data were analyzed using Statistical Package for the Social Sciences software (SPSS) for Windows version 17.0. Descriptive statistics, e.g. frequency distributions and percentage, were calculated. The Chi Square test was used to explore the association between: demographic factors to patterns of DT use and parental approach; patterns of DT use and child behavioral problems; and parental approach and child behavioral problems. Linear regression modeling was used with stepwise variable selection method to find the predictors of child behavioral problems. Before conducting the regression analysis, bivariate analysis was first be done (on the variables related to the characteristics of respondents, their children, their home environment, child’s digital game content and parental approach scores) for identifying potential predictors. Only variables with p-value less than 0.1 were considered in the regression model. The statistical significance for this study was set at the p < 0.05 level.

The scoring methods for the scales, the modified “Parental Approach Patterns on Children’s Media Use” scale is a 4-point Likert scale used for measuring parenting approaches to manage the child’s DT use. The options included ‘Never,’ ‘Seldom’ (1–2 times per week), ‘Sometimes’ (3–4 times per week), and ‘Often’ (5 times or more per week). The ordinal data were converted to interval data for statistical analysis: ‘Never’ as 1, ‘Seldom’ as 2, ‘Sometimes’ as 3, and ‘Often’ as 4 [29]. Average scores of each type of approach were calculated to determine whether the parent adopted a specific type of approach, as the maximum total scores of restrictive approach, instructive approach and co-using approach were 32, 24, and 20 respectively.

A similar 4-point Likert scale was used for the modified “Parent-Child Internet Addiction Test.” The total score for this modified version was 60 and the cut-off point was determined by calculating the proportion of the original version. A score between 15 and 20 points indicates the child has a lower tendency of behavioral problems, while score between 21 and 60 points indicates a higher tendency for behavioral problems due to excessive DT use. The higher the score achieved, the greater the tendency for the child having behavioral problems as an outcome of their DT use.

## Results

### Demographic information

The questionnaires were predominantly completed by mothers (75.7%), fathers (21.8%) and guardians (2.5%). The participating children were 43.6% girls and 56.4% boys. Over 70% of the parents were between the ages of 21 and 40. Nearly half (48%) were stay-at-home parents and 45% were in full-time employment, with 6.4% in part-time employment. Half of the respondent families had incomes between HK$10,000 and HK$29,999 per month (Tables 1 and 2).

**Table 1:** Characteristics of the respondents’ children (N = 202)

**Children’s characteristics**		**Frequency**	**%**
Gender	Male	114	56.4
	Female	88	43.6
Age	3	44	21.8
	4	66	32.7
	5	59	29.2
	6	33	16.3
No. of other children living in the same household	0	79	39.1
	1	76	37.6
	2	27	13.4
	3	10	5.0
	> 3	10	5.0
Siblings	elder brother	42	20.8
	elder sister	44	21.8
	younger brother	30	14.9
	younger sister	32	15.8

**Table 2:** Characteristics of the respondents (N = 202)

**Respondents’ characteristics**		**N**	**%**
Relationship with child	Mother	153	75.7
	Father	44	21.8
	Guardian	5	2.5
Age	<21	0	0
	21-30	43	21.3
	31-40	113	55.9
	41-50	40	19.8
	>50	6	3.0
Education attainment	Primary school	7	3.5
	Secondary school	124	61.4
	Diploma	29	14.4
	Degree	31	15.3
	Post-degree	11	5.4
Employment status	Stay-at-home parent	97	48.0
	Full time employment	91	45.0
	Part time employment	13	6.4
	Job seeking	1	0.5
Type of work	Professional	33	16.3
	Non-professional	42	20.8
	Skilled	21	10.4
	Non-skilled	4	2.0
	Others	4	2.0
	Not working	98	48.5
Family income per month	<$10,000	33	16.3
	$10,000-$19,999	73	36.1
	$20,000-$29,999	29	14.4
	$30,000-$49,999	39	19.3
	>$50,000	28	13.9
Time spent on parent-child DT use	<1 hr	130	64.4
	1-2 hrs	67	33.2
	3-4 hrs	4	2.0
	5-6 hrs	1	0.5

Almost all families (N = 199, 98.5%) had at least 1 computer at home. Around half (54.5%) of the families had computers located in their living room, with the next highest number located in other bedroom (40.1%). Only 16.8% were located in the children’s bedroom. Nearly all families (N = 200, 99.0%) owned at least one television, and 91.1% of the televisions were located in the living room. More than half of the parents (64.4%) spent less than one hour per day together with their children using DTs (Tables 2 and 3).

**Table 3:** Characteristics of respondents’ home environment (N = 202)

**Home environment characteristics**		**N**	**%**
No. of computer(s) at home	0	3	1.5
	1	140	69.3
	2	43	21.3
	3	13	6.4
	4	3	1.5
Location of computers*	Living room	110	54.5
	Other bedroom	81	40.1
	Children’s bedroom	34	16.8
	Study room	36	17.8
	Dining room	9	4.5
No. of television(s) at home	0	2	1.0
	1	132	65.3
	2	57	28.2
	3	9	4.5
	4	2	1.0
Location of televisions*	Living room	184	91.1
	Dining room	19	9.4
	Children’s bedroom	19	9.4
	Other bedroom	56	27.7
	Study room	1	0.4

*Multiple answers are allowed.

### DT Use activities

The top five parent-child DT use activities were sharing pictures, figures and photos (N = 176, 87%), entertainment (N = 132, 65%), searching homework answers (N = 126, 62%), searching other information (N = 122, 60%) and family communication (N = 119, 59%) (See Table 4). The child’s DT use was mainly related to improving cognitive and functional development, which included literacy (N =152, 75%), recognition of shape, sound or color (N = 145, 72%), completing school assignments (N = 118, 58%), and improving memory (N = 118, 58%) (Table 4).

**Table 4:** Parent-child and child online and digital activities involved in last 3 months (N = 202)

**Parent-child online and digital activities**	**N**	**%**
Sharing pictures, figure or photos	176	87.1
For entertainment	132	65.3
Searching homework answers	126	62.4
Searching for other information	122	60.4
Family communication	119	58.9
Searching for health related information	113	55.9
For news	107	53.0
For enquiries and questions	90	44.6
Talking with friends	82	40.6
Sending messages	75	37.1
Seeking supplier and purchasing	70	34.7
Getting friends	40	19.8
**Child online and digital activities**		
For literacy	152	75.2
Recognition of shape, sound or colors	145	71.8
Completing school assignment	118	58.4
Improving memory	118	58.4
Story telling	116	57.4
Improving eye-hand coordination	115	56.9
For enjoyment and fun	113	55.9
For calculations	111	55.0
Improving reaction and response	111	55.0
Improving fine motor coordination	107	53.0
Improving attention span	102	50.5
Drawing	99	49.0
For sciences	74	36.6
Social networking	36	17.8

DT use patterns were significantly related to a child’s age and gender, parent’s age, number of children in the family, parental employment status, family income, and location of the computer. Three main areas were identified for DT use: education, entertainment and communication. In the area of education related to searching for homework answers, DTs were used more frequently by older preschool children than younger children (*x*^2^ = 6.31, *p* < 0.05); stay-at-home parents than working parents (*x*^2^ = 3.99, *p* < 0.05); and children with lower family incomes than those with higher family income (*x*^2^ = 6.67, *p* < 0.05). When completing assignments, DTs were used more frequently by older preschool children than younger children (*x*^2^ = 10.41, *p* < 0.01); children with a lower family income than those with a higher family income (*x*^2^ = 6.74, *p* < 0.01). For browsing the news, DTs were used more frequently by stay-at-home parents than working parents (*x*^2^ = 6.57, *p* < 0.05). In regard to entertainment, DTs were used more frequently by children with younger parents (<30 years old) (*x*^2^ = 6.21, *p* < 0.05); and children who have computers located in other bedrooms (*x*^2^ = 4.60, *p* < 0.05). Families with two or more children used DTs more frequently for talking with friends than those with only one child (*x*^2^ = 4.03, *p* < 0.05).

### Content of digital games

Parents reported that their children’s digital games contained the visuals of aggressive behavior (Animated: N = 63, 31.2%; Real person: N = 10, 5%); antisocial behavior (Animated: N = 58, 28.7%; Real person: N = 14, 6.9%); unrealistic expectation (Animated: N = 43, 21.3%; Real person: N = 10, 5%); and risky behavior (Animated: N = 19, 9.4%; Real person: N = 12, 5.9%) in animated cartoons and photographs of real person in action (Table 5).

**Table 5:** Child’s digital game content (N = 202)

**Content**	**Features of digital games**	**N**	**%**
Aggressive behavior	Animated (Cartoon)	63	31.2
	Photographs of real person in action	10	5.0
Antisocial behavior	Animated (Cartoon)	58	28.7
	Photographs of real person in action	14	6.9
Unrealistic expectation	Animated (Cartoon)	43	21.3
	Photographs of real person in action	10	5.0
Risky behavior	Animated (Cartoon)	19	9.4
	Photographs of real person in action	12	5.9

Note: Examples of the contents. 1) Aggressive behavior: Presence of fighting, boxing, bloody scene, killing; 2) Antisocial behavior: Offensive language, rude humor, tantrum, uncontrolled scream; 3) Unrealistic expectation: Money winning, gold digging, super powers; and 4) Risky behavior: Presence of smoking/drug use/alcohol use, reckless driving.

### Parenting approach

The highest mean score among the three parenting approaches was used to determine the main parenting approach. The percentage of parents adopting an ‘instructive mainly’ approach of preventing their children’s excessive usage of DT was 45.5% (N = 92), with mean instructive approach score equal to 3.13 (SD = 0.55). The percentage of parents adopting a ‘co-using mainly’ approach was 22.8% (N = 46) with mean co-using approach score equal to 2.93 (SD = 0.62), and the percentage of parents using ‘restrictive mainly’ approach was 18.3%, (N = 37) with mean restrictive approach score equal to 2.86 (SD = 0.70) (Table 6). There was no significant relationship found between demographic characteristics, educational level, number of children in the family and the three parenting approaches.

**Table 6:** Usage pattern of different types of parenting approach (N = 202)

	**N**	**%**	**Mean score (SD)**		
			**Instructive**	**Co-using**	**Restrictive**
Instructive mainly^#^	92	45.5	3.13 (0.55)	2.63 (0.56)	2.70 (0.53)
Co-using mainly^#^	46	22.8	2.56 (0.64)	2.93 (0.62)	2.27 (0.65)
Restrictive mainly^#^	37	18.3	2.50 (0.73)	2.35 (0.68)	2.86 (0.70)
All three approaches	13	6.4	1.85 (0.90)	1.85 (0.90)	1.85 (0.90)
Instructive & co-using	9	4.5	3.11 (0.78)	3.11 (0.78)	2.76 (0.70)
Restrictive & instructive	5	2.5	2.70 (0.76)	2.28 (0.30)	2.70 (0.76)
Co-using & restrictive	0	0.0	-	-	-

^#^highest mean score was used to determine the main parenting approach.

### DT related behavioral problems

Behavioral problems were identified as related to DT use. Children had a higher tendency for developing behavioral problems when using DT in searching homework answers (*x*^2^ = 5.57, *p* < 0.05), completing school assignments (*x*^2^ = 4.90, *p* < 0.05), interacting with animated cartoons that contained aggressive behavior (*x*^2^ = 5.34, *p* < 0.05) and antisocial behavior (*x*^2^ = 10.75, *p* < 0.01).

The result of linear regression in Table 7 showed that the ‘restrictive approach score’ was the only predictor among the three parental approaches; suggesting that the frequent adoption of a restrictive approach will increase the children’s behavioral problem score (B:1.66, 95% CI:[0.21,3.11], p < 0.05). Children viewing antisocial behavior cartoons significantly increased the tendency of children’s behavioral problems (B:3.84, 95% CI:[1.66, 6.02], p < 0.01). Although the factor of total time spent with the child on online and digital activities was also retained in the regression model, the factor was insignificant (B:1.44, 95% CI:[-0.17, 3.04], p = 0.08).

**Table 7:** Linear regression model on children’s behavioral problem (N = 202)

**Predictors**	**B**	**95% CI**	**p-value**
Viewed antisocial behavior cartoon	3.84	(1.66, 6.02)	0.001**
Total time spent with child on online and digital activities	1.44	(-0.17, 3.04)	0.08
Restrictive approach score	1.66	(0.21, 3.11)	0.025*

Dependent Variable: Behavioral problem score; *p < 0.05; **p < 0.01; The following items were considered but finally removed by the stepwise variable selection method from the regression model: viewed aggressive behavior cartoon; viewed aggressive behavior photographs of real person in action; viewed antisocial behavior photographs of real person in action; viewed risky behavior cartoon; instructive approach score; and co-using approach score.

## Discussion

Parents play a crucial role in promoting and maximizing their preschool children’s healthy DT use. As preschoolers will try various ways to explore their world [28] to maximize crucial experiences, a balance is necessary between DT use and the performance of daily.

This study has investigated DT use patterns and behaviors of Hong Kong preschool children and their parents, and the relationship to three parenting approaches—restrictive, instructive and co-using. Several issues were identified within this study including the time preschoolers spent using DTs, and the link between the type of parenting approach and behavioral problems.

As easier access to DTs becomes the norm, it is not surprising that nearly all families studied owned at least one computer. This indicated that Hong Kong preschool children are likely to be regularly exposed to DTs. The accessibility rate for computers has increased by about 20 percentage points in the past decade [17]. These results were consistent with the findings of the North American study carried out by Rideout [30] who identified that 98.0% of preschool children’s families had at least one television at home. Moreover, it was found that more than half of the computers at home were located in bedrooms or study rooms; this is consistent with studies in other countries that demonstrate an increase in location and use of DTs in separated rooms [21]. The location of the DT use is critical to the ability of parents to actively monitor and supervise their preschooler’s use of televisions, computers and other DTs.

In this study, over half of the parents spent less than one hour per day interacting with their children when using DT. This amount of time spent using DTs by these preschool children is within the recommended screening time of children of less than 2 hours a day [19]. Neuroscience research demonstrates that DT use experience of young children has the potential to shape the structures and long term functioning of the brain [31].

It was not surprising that in this study around 60% of parents used DTs for educational purposes, for example, searching homework answers. According to Plowman and Stephen [32], preschool children are commonly encouraged to use DTs for educational purposes as many parents believe that DT use can facilitate an increase in childhood cognitive, functional and social development, and enhance academic school achievement. This parental behavior may be explained in light of Chinese culture as academic achievement is identified as a stepping stone for future success, as well as a reflection of “good” parenting [22,33].

Parents have a significant role in preschoolers’ DT use management. In Warren’s [34] examination of parental approach to managing preschool children’s (ages 1 to 5) television viewing, it was suggested that parents set more rules for their younger children than school-aged children or adolescents. This has some similarities with the findings in this study, in which it was found that many parents practiced restrictive approaches to DT use, in combination with instructive and co-using parenting approaches. It is reasonable to conclude that the use of rules increases during the preschool years, as parents want to protect children from exposure to unhealthy media content and other risks. Therefore, restrictive approach exerted greater effects on children with lower self-control and more supports for children to develop self-regulation through setting rules that guide their behaviors [35]. However, imposing too many restrictions on preschool children has the potential to result in an opposite effect of the children desiring more independent use, resulting in increased negative behavior [35].

It is worth noting that a restrictive parenting approach was often used in combination with either an instructive or co-using approach. This is understandable given the complexity of parenting a preschooler. When using an instructive approach, it has been identified that the ensuing discussion enhances children’s critical thinking development and causes them to be more critical about media content [36]. However, preschoolers are more interested in simply labeling objects and actions seen in media; rather than a complex discussion about the characters, plots, and motives [34]. Too much discussion on the content may hinder the use for exploration during DT use, leading to negative outcomes.

Preschool children are not cognitively mature enough to be responsible for self-determining the content of DTs [37,38]. This is supported by the findings that parent-child DT use with content of aggressive behavior and antisocial behaviors are found to have a higher tendency to behavioral problems than those who did not [39,40]. Repetitive and redundant portrayals of unwanted behaviors may prompt children to develop expectations, perceptions and thoughts of those behaviors.

### Limitations

This study had several limitations. The first was the size of the targeted group and the use of a convenience sample, with parents self-selecting to participate in the study. This sample may not have been representative of the Hong Kong parent population of preschool children. A large proportion of the recruited subjects were mothers and thus the associations drawn that related to gender are not conclusive.

The second limitation was the possibility of a family having more than one preschool child. This potentially resulted in conflation of a parent’s responses of two or more of their children’s behaviors and their responses to the situation. The third limitation was the potential for parents to over or underestimate the frequency of their child’s behavioral problems during DT use, by requiring the parent to reflect on past events resulting in subjective responses. This study did not include items related to child’s temperament, attachment status or parenting stress. The fourth limitation was the differentiation of different kinds of DT product, such as television, digital tablet, and smart phone. A more detailed study is needed with the information of each specific type of devices. The final limitation was the ever-present risk that parents would provide a socially desirable response even though they were informed of questionnaire anonymity and confidentiality.

While this quantitative study has identified some factors affecting parent-child DT use, the use of a qualitative method that includes ethnographic techniques of observation would add more depth to this study. In addition, it would be worthwhile to investigate whether demographic factors, and the amount of time children spent with DTs alone have a direct influence on a child behavior as well as the association between DT use patterns and selected combinations of parental approaches.

## Conclusions

DTs are widely available and regularly accessed by families and their preschool children as an accepted part of daily life. This raises the need for parents to monitor their children’s DT use to minimize potential health and development. Excessive DT use has ramifications for the emotional, psychological and social aspect of a child’s everyday life, affecting their family and school life. While parents play an important role in influencing children’s appropriate DT use, positive outcomes on children DT use resulted only with appropriate parental mediation and modeling.

Since preschool children’s cognitive and functional abilities are still in the developmental stage, parents play a crucial role in fostering appropriate and safe DT use. It is suggested that parents practice a combination of restrictive, instructive and co-using approaches, rather than a predominately restrictive approach, in order to facilitate their child’s growth and development. Further studies are needed to explore the parent-child relationship and parents’ self-efficacy when managing the parent-child DT use, in order to develop strategies to guide children in healthy DT use.

## Abbreviations

DT: Digital technology.

## Competing interests

The authors declare that they have no competing interest.

## Authors’ contributions

CSTW, CF, AYL, WYYL: participated in the design, CSTW, CHMW in data collection; CSTW, CHMW, HTW: analysis of data; CSTW, CF, HTW, AYL: discussion; CSTW, CF, AYL, HTW, WYYL: drafted the manuscript; All of the authors read and approved the final version of the manuscript.
